# PPLA-Transformer: An Efficient Transformer for Defect Detection with Linear Attention Based on Pyramid Pooling

**DOI:** 10.3390/s25030828

**Published:** 2025-01-30

**Authors:** Xiaona Song, Yubo Tian, Haichao Liu, Lijun Wang, Jinxing Niu

**Affiliations:** School of Mechanical Engineering, North China University of Water Resources and Electric Power, Zhengzhou 450045, China; songxiaona1@126.com (X.S.); 201721908@stu.ncwu.edu.cn (Y.T.); liuhaichao@ncwu.edu.cn (H.L.); niujinxing@ncwu.edu.cn (J.N.)

**Keywords:** defect detection, transformer, attention

## Abstract

Defect detection is crucial for quality control in industrial products. The defects in industrial products are typically subtle, leading to reduced accuracy in detection. Furthermore, industrial defect detection often necessitates high efficiency in order to meet operational demands. Deep learning-based algorithms for surface defect detection have been increasingly applied to industrial production processes. Among them, Swin-Transformer achieves remarkable success in many visual tasks. However, the computational burden imposed by numerous image tokens limits the application of Swin-Transformer. To enhance both the detection accuracy and efficiency, this paper proposes a linear attention mechanism based on pyramid pooling. It utilizes a more concise linear attention mechanism to reduce the computational load, thereby improving detection efficiency. Furthermore, it enhances global feature extraction capabilities through pyramid pooling, which improves the detection accuracy. Additionally, the incorporation of partial convolution into the model improves local feature extraction, further enhancing detection precision. Our model demonstrates satisfactory performance with minimal computational cost. It outperforms Swin-Transformer by 1.2% mAP and 52 FPS on the self-constructed SIM card slot defect dataset. When compared to the Swin-Transformer model on the public PKU-Market-PCB dataset, our model achieves an improvement of 1.7% mAP and 51 FPS. These results validate the universality of the proposed approach.

## 1. Introduction

Surface defects on industrial products, such as scratches, cracks, stains, and chippings, not only affect the appearance of quality of the products but also reduce their service life and may even affect their safety performance. Therefore, it is crucial to perform defect detection on industrial products. With the continuous advancements in computer technology and deep learning, the application of machine vision for the detection of surface defects has increasingly emerged as a widely adopted method [1,2]. Since the introduction of AlexNet [3] in 2012, a myriad of object detection models based on convolutional neural networks (CNNs) [4,5,6,7] have been developed. These models demonstrate remarkable performance across various visual tasks. CNNs primarily enhance contextual information extraction by increasing the number of layers and utilizing larger convolutional kernels to expand the receptive field. However, they predominantly concentrate on local feature extraction, which poses challenges in directly capturing long-distance global contextual information.

In recent years, Transformers [8] have increasingly been employed in many visual tasks [9,10,11], demonstrating their remarkable potential. Nevertheless, the high-resolution images encompass a pixel count that significantly surpasses the number of tokens that Transformers are capable of processing. Moreover, the complexity of the multi-head self-attention mechanism in Transformers is proportional to the square of the number of input tokens. The escalating computational demand presents substantial challenges for existing resources, thereby limiting the effectiveness of Transformers in visual tasks. Some researchers have revised their methodologies by subdividing images into smaller segments. Others utilize compact feature maps derived from convolutional processes. These approaches aim to decrease the number of input tokens utilized in their analyses. While these initiatives have yielded some advancements, they simultaneously diminish the remarkable contextual extraction abilities of Transformers. Among these methods, Swin-Transformer [12] constrains the computation of the self-attention mechanism to a limited window. And it acquires contextually relevant information through a shifting window approach. This approach achieved significant success during that period. However, the intricate nature of the multi-head self-attention mechanism continues to constrain both the training and inference processes of the Swin-Transformer in certain application contexts. The Pyramid Pooling Transformer (P2T) [13] introduces a pyramid pooling technique designed to reduce the sequence length while simultaneously enhancing the extraction of contextual features. The Efficient Vision Transformer (EfficientViT) [14] introduces a novel approach by substituting the computationally intensive softmax attention mechanism with a more straightforward ReLU linear attention. This adaptation enables the model to achieve a global receptive field while maintaining efficiency without incurring loss. Both of these approaches are highly effective in enhancing the efficiency of the Transformer’s multi-head self-attention mechanism. Therefore, this paper integrates the advantages of two methods to enhance the multi-head self-attention mechanism. Specifically, we employ a more simplified and efficient linear attention based on pyramid pooling in place of the traditional multi-head self-attention mechanism utilized by Swin-Transformer. Additionally, we incorporate partial convolution [15] to enhance the extraction of local information, thereby making the model pay more attention to the defect-related details. Our main contributions are outlined as follows:

1. We propose a linear attention mechanism based on pyramid pooling (PPLA). It integrates pyramid pooling with multi-scale linear attention, significantly enhancing the efficiency of Transformers. This approach not only conserves substantial computational resources but also facilitates training and inference processes. Furthermore, through the implementation of partial convolution, the model not only achieves an efficient global receptive field but also demonstrates a remarkable ability to accurately capture local defect information, thereby significantly enhancing detection accuracy.
2. We apply PPLA to the Swin-Transformer and construct an efficient model to detect the defects in industrial products. At the same time, we evaluate its performance on our self-built SIM card slot dataset as well as the publicly available PKU-Market-PCB dataset. The results demonstrate that the method we propose not only enhances the accuracy of defect detection but also significantly improves detection efficiency.

The remainder of this paper is organized as follows. In Section 2, we introduce the relevant literature on defect detection and explain our motivation for proposing the PPLA-Transformer. Section 3 provides a comprehensive overview of the details pertaining to the PPLA-Transformer. The results of our experiments are presented in Section 4. Finally, in Section 5, we summarize the contributions and limitations of our work, as well as outlining potential directions for future research.

## 2. Related Work

The manual detection of defects is costly and inefficient. Consequently, in industrial production, the visual inspection of product appearance defects is increasingly being supplanted by machine vision techniques [16,17]. In recent years, the advent of deep learning theory has ushered in a new era for defect detection. Deep learning-based methods have emerged as a highly efficient and innovative solution. They are particularly effective in identifying surface defects in industrial products. Researchers have improved general object detection frameworks to meet the specific detection requirements of industrial data.

CNN architectures are used in early object detection techniques. There are two-stage object detection frameworks based on region proposal networks, such as R-CNN and its variants [18,19,20], as well as one-stage object detection frameworks exemplified by the YOLO series [21,22,23]. These models use anchor boxes to integrate object classification with localization regression, thereby optimizing the detection speed of the models. Cha et al. [24] identify bridge defects using the two-stage detector Faster R-CNN [19] and enhance the real-time performance by replacing the backbone network with ZF-net. Li et al. [25] employ the one-stage detector YOLO to identify surface defects in steel, incorporating shallow features to enhance the detection capabilities for minor imperfections. Chen et al. [26] integrate DenseNet [27] with YOLOv3 for the detection of LED defects. There are also some studies on the lightweight nature of models to improve the efficiency of CNNs, including the MobileNet series [28,29,30], ShuffleNets [31,32], EfficientNets [33,34], and so on. FasterNet [15] introduces a partial convolution technique aimed at improving efficiency by decreasing redundant computations and memory accesses.

In 2017, Google proposed the Transformer [8] model, which captures contextual information in input text data through self-attention mechanisms. This innovation has achieved remarkable success in the realm of natural language processing. Since then, many approaches based on the Transformer architecture have emerged. The Vision Transformer (ViT) [11] introduces the Transformer architecture into the field of computer vision (CV). The ViT model surpasses the performance of state-of-the-art CNNs on the ImageNet dataset [35]. Gao et al. [36] introduce a modified version of the Swin-Transformer, known as Cas-Swin, specifically designed for the detection of surface defects on rollers. Zhou et al. [37] propose ETDNet, which integrates CM-FPN into the Transformer architecture to effectively identify surface defects in steel materials. Wang et al. [38] propose the Defect Transformer (DefT), which integrates the benefits of Local Positional Awareness Blocks (LPBs), Lightweight Multi-Pooling Self-Attention (LMPS), and Convolutional Feedforward Networks (CFFNs). This innovative approach demonstrates its performance in the defect detection of various material surfaces, including steel, fabric, and so on. However, owing to its intricate attention mechanism, the ViT needs a substantial quantity of annotated data for training. This requirement is often impractical for defect detection tasks that prioritize efficiency and lightweight deployment. DeiT [39] utilizes knowledge distillation methods alongside a range of data augmentation techniques to decrease the computational resources necessary for training ViTs. CvT [40] integrates deep convolution into the self-attention mechanism of Transformers by employing convolutional projections to compute the queries (Q), keys (K), and values (V). Notably, a stride of two is applied in the convolutional projections for computing K and V, which effectively reduces the computational parameters while preserving equivalent performance. T2T-ViT [41] endeavors to establish some degree of overlap when partitioning the image into smaller blocks. This approach enhances the local contextual relationships among the image blocks. However, it does not entirely resolve the issue of objects being divided by different image segments. Swin-Transformer uses a shifting window approach to divide image blocks, limiting the calculation of the self-attention mechanism to smaller image blocks. And that brings better context and multi-scale information by expanding the image blocks gradually. Therefore, Swin-Transformer is used as our baseline architecture in this paper.

Furthermore, there are research studies that utilize pooling methods to augment the capabilities of Vision Transformers. The Pyramid Vision Transformer (PVT) [42] and the Multi-Scale Vision Transformer (MViT) [43] implement single-layer pooling to reduce the number of tokens required for the multi-head self-attention mechanism in Transformers. The P2T [13] proposes a multi-head self-attention mechanism based on pooling, which is very simple and efficient. However, the self-attention mechanism employed by Transformers still requires significant computational resources due to the softmax-based exponential calculations. The EfficientViT [14] introduces a multi-scale linear attention mechanism. It replaces the complex softmax computations with a simplified ReLU linear attention approach, which yields competitive performance on various tasks and datasets.

The efficiency of detection is crucial for the inspection of industrial products. Directly applying existing models to the defect detection of industrial products is often impractical. Many models can attain higher detection accuracy. However, the inspection efficiency remains low. Although some models assert their capability to balance both inspection efficiency and accuracy, they often fail to meet the efficiency requirements which is necessary for effective industrial defect detection. Thus, we propose a linear attention mechanism based on pyramid pooling (PPLA), which effectively reduces the computational demands of the multi-head self-attention mechanism through pyramid pooling. Additionally, we replace softmax attention with ReLU linear attention to further reduce the computational burden and improve the efficiency of defect detection. Because the linear attention mechanism may compromise the ability to extract local features, we enhance local feature extraction by employing partial convolution. Our proposed method preserves the excellent global modeling capabilities of the Transformer. It effectively captures tiny defect information, leading to a significant enhancement in defect detection accuracy. Ultimately, our model strikes an optimal balance between accuracy and efficiency in the realm of industrial defect detection.

## 3. Method

In this section, we first introduce the fundamental principles of PPLA. Then, we explain the PPLA-Swin block which is the primary module in our model. Finally, the structure of the PPLA-Transformer model is detailed.

### 3.1. PPLA

**Figure 1:**
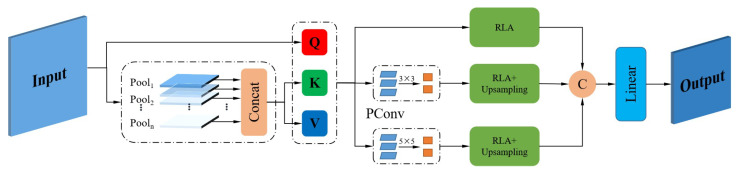
The structure of PPLA. “${\mathrm{Pool}}_{1},{\mathrm{Pool}}_{2},\ldots ,{\mathrm{Pool}}_{n}$” refers to the maximum pooling operation performed at various scales. This method enables the extraction of the most significant features within a region across different scales. “PConv” refers to partial convolution, which consolidates adjacent feature tokens in order to generate multi-scale feature maps. “RLA” denotes ReLU linear attention, which substitutes the complex exponential computations of the softmax function with a more straightforward ReLU function. This modification significantly reduces the computational burden of the model while preserving its performance.

As shown in Figure 1, PPLA integrates pyramid pooling with multi-scale linear attention which can fuse multi-scale features and obtain a global receptive field with little computational cost so as to obtain faster detection speed and better detection results.

First, the input images are respectively passed through multiple maximum pooling layers of different scales to extract the most significant features at different scales, and the formula is presented as follows:

(1) $$\begin{array}{cc} P_{1} & ={\mathrm{MaxPool}}_{1}\left(X\right)\\ P_{2} & ={\mathrm{MaxPool}}_{2}\left(X\right)\\ \vdots \\ P_{n} & ={\mathrm{MaxPool}}_{n}\left(X\right) \end{array}$$

where ${P_{1},P_{2},\ldots ,P}_{n}$, represents the feature maps generated by the maximum pooling operation, n denotes the number of maximum pooling layers applied, and MaxPool refers to the maximum pooling process. Selecting an appropriate pooling rate can make the size of the feature map ${P_{1},P_{2},\ldots ,P}_{n}$ obtained by the maximum pooling smaller than the input X. Since maximum pooling is a linear computation, the above process requires few computational resources. Subsequently, the feature maps are concatenated, and layer normalization is carried out then. The formula is

(2) $${P=\mathrm{LayerNorm}(\mathrm{Concat}(P_{1},P_{2},\cdots ,P}_{n}))$$

where P denotes the feature maps obtained after fusion. LayerNorm is a layer normalization process. The feature maps P not only contain the most significant feature information of input X at different scales but also contain rich context information. The formula for Q, K, and V is as follows:

(3) $$\left(Q,K,V\right)=\left({\mathrm{XW}}^{q},{\mathrm{PW}}^{k},{\mathrm{PW}}^{v}\right)$$

where $W_{q},W_{k},\mathrm{and}\;W_{v}$ represent the weight matrix of linear transformation, respectively. When generating K and V, we replace the input X with P, which uses fewer parameters while retaining rich feature information. Next, we adopt the ReLU linear function to implement a linear attention mechanism. The similarity function is expressed as follows:

(4) $$\begin{array}{c} \mathrm{sim}\left(Q,K\right)=\mathrm{ReLU}\left(Q\right)\mathrm{ReLU}{\left(K\right)}^{T}\\ \mathrm{ReLU}\left(X\right)=\left\{\begin{array}{cccccc} \; & 0\; & ,\; & X & < & 0\\ \; & X\; & ,\; & X & > & 0 \end{array}\right. \end{array}$$

Therefore, the attention mechanism can be written as

(5) $$\mathrm{Attention}\left(Q,K,V\right)=\sum_{i=1}^{N}\frac{\mathrm{ReLU}\left(Q\right)\mathrm{ReLU}{\left(K_{i}\right)}^{T}}{\sum_{i=1}^{n}\mathrm{ReLU}\left(Q\right)\mathrm{ReLU}{\left(K_{i}\right)}^{T}}V_{i}$$

According to the associative law of matrix multiplication, the formula presented above can be reformulated as follows:

(6) $$\begin{array}{cc} \mathrm{Attention}(Q,K,V) & =\sum_{i=1}^{N}\frac{\mathrm{ReLU}\left(Q\right)\mathrm{ReLU}{\left(K_{i}\right)}^{T}}{\sum_{i=1}^{n}\mathrm{ReLU}\left(Q\right)\mathrm{ReLU}{\left(K_{i}\right)}^{T}}V_{i}\\ \; & =\frac{\mathrm{ReLU}\left(Q\right)(\sum_{i=1}^{N}\mathrm{ReLU}{\left(K_{i}\right)}^{T}V_{i})}{\mathrm{ReLU}\left(Q\right)(\sum_{i=1}^{N}\mathrm{ReLU}{\left(K_{i}\right)}^{T})} \end{array}$$

There is no doubt that the complexity of linear attention is much smaller than the exponential complexity of the softmax function, which is essential to improve the overall computational efficiency of the model. Furthermore, it enhances the model’s ease of training, prediction, and deployment on more devices.

The linear attention mechanism uses a simple ReLU linear function to replace the complex softmax function, achieving amazing efficiency. However, it has some limitations. Due to the lack of nonlinear characteristics, the linear attention mechanism cannot suppress the output probability corresponding to the non-target category like the softmax attention mechanism. This may result in the model’s inadequate ability to accurately identify the region that requires attention, potentially leading to erroneous judgments and inappropriate focuses on incorrect areas. Therefore, we employ the partial convolution to address this issue. As illustrated in Figure 1, the PConv module employs feature tokens adjacent to partial convolution aggregation to construct multi-scale feature maps. This approach facilitates the linear attention mechanism in enhancing local information. After acquiring multi-scale feature tokens, the ReLU linear attention (RLA module illustrated in Figure 1) is employed for comprehensive global feature extraction. It is followed by the up-sampling and concatenation of multi-scale feature information. Ultimately, the final output is generated through a linear projection layer.

**Figure 2:**
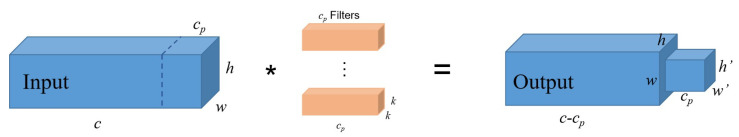
Partial convolution. The continuous channels $c_{p}$ of the input feature acquire new features through *n* convolutional kernels. These newly obtained features are then fused with the features from the remaining *c*-$c_{p}$ channels, contributing to subsequent calculations.

Since the feature maps of most channels in traditional convolution kernel have similar features, it is possible to select a subset of channels that effectively represents the entire feature map. Partial convolution employs a standard convolution kernel on specific channels of input, while maintaining the integrity of the rest channels. Usually the first or last continuous channels are chosen to represent the entire feature map for calculation. As shown in Figure 2, for a feature map with an input size of h×w×c, *n* convolution kernels of dimension $k\times k\times c_{p}$ are applied to the first $c_{p}$ continuous channels, while the remaining *c*-$c_{p}$ channels remain unchanged. Then, the results of the first $c_{p}$ channels are amalgamated with the remaining channels for subsequent calculations. The partial convolution method is employed to aggregate nearby feature tokens in order to construct multi-scale feature maps. Additionally, the model leverages the exceptional local feature extraction capabilities of the CNN, thereby enhancing its overall feature extraction performance while maintaining little computational overhead.

### 3.2. PPLA-Swin Block

We apply PPLA to the Swin-Transformer and propose the PPLA-Swin module. The Swin-Transformer, which applies self-attention to shifting windows, offers an improved context. In contrast, utilizing self-attention on fixed windows remains effective for extracting local features from small defect targets.

**Figure 3:**
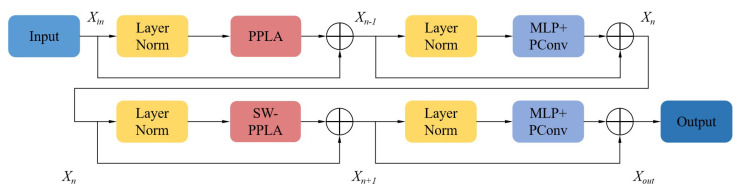
PPLA-Swin block. “LayerNorm” refers to a layer normalization operation, “PPLA” is a linear attention mechanism based on pyramid pooling introduced in Section 3.2, “SW-PPLA” is the “PPLA” of the shifting window, “MLP” represents a multi-layer perceptron, “PConv” is partial convolution, and “MLP” and “PConv” jointly constitute a feedforward neural network.

In the Swin-Transformer, the self-attention on shifting windows brings better context information, and the self-attention on fixed windows is effective for extracting local features of small defect targets. Therefore, we also implement PPLA in both fixed-size and shifting windows. Figure 3 shows the overall composition of the PPLA-Swin module. The PPLA-Swin module consists of two similar parts, distinguished by the windows being fixed or shifting. The input passes through the PPLA module with layer normalization, the FFN module (which is essentially an MLP with PConv), the SW-PPLA module with layer normalization, and the FFN module subsequently. During this process, the output of each module is added to its own input via a residual connection. The above process can be expressed as

(7) $$\begin{array}{cc} \; & X_{n-1}=\mathrm{PPLA}(\mathrm{LayerNorm}\left(X_{in}\right))+X_{in}\\ \; & X_{n}=\mathrm{FFN}(\mathrm{LayerNorm}\left(X_{n-1}\right))+X_{n-1}\\ \; & X_{n+1}=\mathrm{SW}-\mathrm{PPLA}(\mathrm{LayerNorm}\left(X_{n}\right))+X_{n}\\ \; & X_{out}=\mathrm{FFN}(\mathrm{LayerNorm}\left(X_{n+1}\right))+X_{n+1} \end{array}$$

where the input and output of the PPLA-Swin block are denoted as $X_{in}$ and $X_{out}$, respectively, the outputs of the PPLA and the SW-PPLA are represented by $X_{n-1}$ and $X_{n+1}$, respectively, and $X_{n}$ denotes the output from the first FFN module.

PPLA module can reduce computation and maintain the connection of the local context information, while SW-PPLA enhances the model’s capability to capture global context information by incorporating a shifting window mechanism. The combination of the two modules enables the PPLA-Swin module to process high-resolution defect images efficiently while maintaining computational efficiency and model performance.

### 3.3. PPLA-Transformer Model

**Figure 4:**
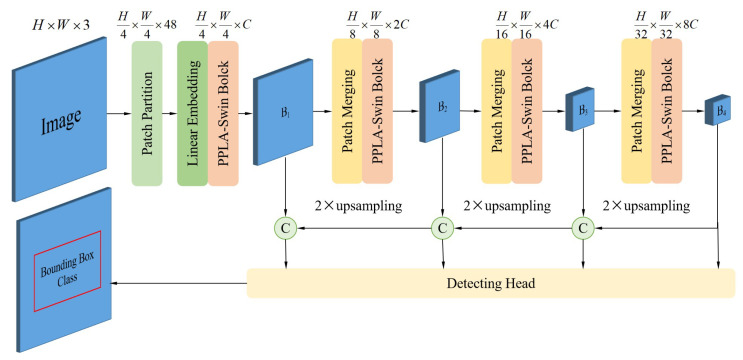
The structure of PPLA-Transformer model. The “Patch Partition” segments the input image into small, non-overlapping sections. The “Linear Embedding” projects the two-dimensional image data onto a token data to facilitate processing by Transformers. The “PPLA-Swin Block” is described in Section 3.2; $B_{1},\;B_{2},\;B_{3},\;B_{4}$ refer to the feature map corresponding to stages 1, 2, 3, and 4.

Based on the PPLA-Swin module, we construct a PPLA-Transformer model to complete the defect detection. Figure 4 illustrates the architecture of the PPLA-Transformer model. Our model is divided into four stages. In the first stage, the Patch Partition segments the input image into distinct patches, while the Linear Embedding layer maps these patch images to a desired dimension, denoted as *C*. This transformation allows the two-dimensional image data to be converted into a format that is amenable for processing by Transformer architectures. Then, the PPLA-Swin module is followed by the Linear Embedding layer to obtain the results of the first stage. The second, third, and fourth stages are composed of a down-sampling layer and a PPLA-Swin module. Moreover, the feature maps of the three stages are fused with the feature maps of the previous stage after 2× up-sampling. Concurrently, we perform predictions on the fused feature maps derived from the outputs at each stage. In this way, the model can make use of both the accurate location information on the small-size feature map and the rich semantic information on the large-size feature map to realize the accurate location and classification of the defect location.

## 4. Experiment

### 4.1. Dataset

**Figure 5:**
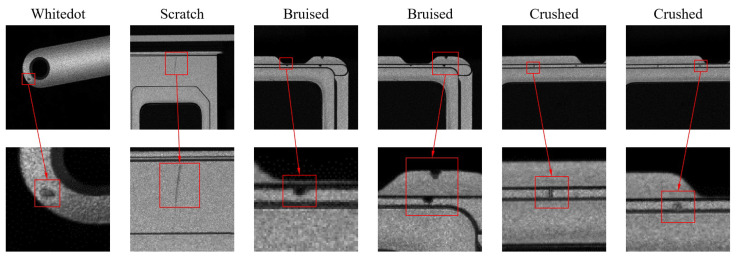
Some images from the self-built SIM card slot surface defect dataset (**top**) and their corresponding zoomed-in defect details (**bottom**).

In this study, a dataset of 3,655 surface defect images from SIM card slots is collected. The defects include whitedot, scratched, bruised, and crushed, with each type comprising 22%, 27%, 25%, and 26% of the total, respectively. Figure 5 presents the original images of each defect (top) and zoomed-in details (bottom). The dataset is then expanded to 7310 images using data augmentation techniques. The images are labeled and saved in VOC format using LabelIMG software of version 1.8.6, and subsequently converted to COCO format via a conversion script. Finally, the dataset is partitioned into training, validation, and test sets in an 8:1:1 ratio.

**Figure 6:**
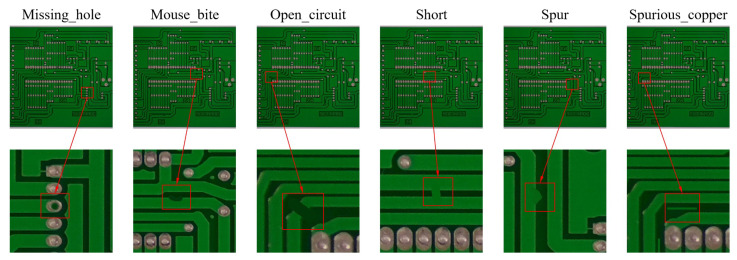
Some images from the PKU-Market-PCB dataset (**top**) and their corresponding zoomed-in defect details (**bottom**).

Additionally, we validate our improvements on the PKU-Market-PCB dataset released by Peking University. This dataset consists of 1386 images representing six types of defects: missing hole, mouse bite, open circuit, short, spur, and spurious copper. Figure 6 illustrates representative images of each defect in the PKU-Market-PCB dataset (top) and zoomed-in details (bottom).

### 4.2. Experimental Environment and Setup Details

The experiments are conducted in a Windows environment, with Python version 3.8.0 and PyTorch version 1.8.2. Training is accelerated using an RTX A5000 GPU with CUDA version 11.1.

AdamW is employed as the optimizer, with an initial learning rate of 0.001 and a weight decay of 0.05. The batch size is set to 16 images, and the model is trained for 300 epochs. During each epoch, the model is trained on the training set, and after each training step, the validation set is used to guide the gradient descent process for convergence. After 300 epochs, the test set is used to assess whether the model had overfitted to the training data. The image size for training, validation, and testing is adjusted to 224×224 pixels.

### 4.3. Experimental Results and Analysis

#### 4.3.1. Experiments on the Self-Built SIM Card Slot Defect Dataset

Experiments are conducted on the self-built SIM card slot defect dataset using Faster R-CNN, YOLO-v8, P2T, EfficientViT, Swin-Transformer, and PPLA-Transformer (Ours). The results are shown in Table 1. We use the model’s mean Average Precision (mAP) at an Intersection over Union (IoU) threshold of 0.5 on the test set, and Frames Per Second (FPS) during inference, as well as the floating point of operation (FLOP) as comparison metrics. All models are evaluated with identical parameter settings and data preprocessing methods. Our model demonstrates a prominent advantage over mainstream object detection models in both mAP and FPS. In terms of FLOPs, our model has a 4.3 G reduction compared to the original Swin-Transformer. Due to the reduced computational load, our model is significantly faster than the original Swin-Transformer and other models in terms of inference speed. At the same time, our model also remains ahead in mAP. Specifically, our model outperforms the original Swin-Transformer by 1.2% in mAP and achieves a 52 FPS improvement in detection speed, significantly surpassing the original Swin-Transformer and other models.

Specifically, as shown in Table 1, compared to the newer object detection models, Faster R-CNN exhibits an uncompetitive performance in both mAP and FPS. YOLO-v8 performs at an intermediate level in terms of both detection accuracy and speed. The Swin-Transformer demonstrates strong competitiveness in detection accuracy. However, due to the computational complexity of the Transformer self-attention mechanism, the Swin-Transformer struggles with detection speed. Although P2T and EfficientViT reduce the computational complexity of the Transformer to some extent, their detection accuracy is slightly lower. Moreover, their detection speeds do not show a clear advantage over the YOLO series. Our model significantly reduces the computational complexity of the Transformer by employing a pyramid pooling-based linear attention mechanism. Additionally, by applying pyramid pooling to the original images, our model captures more contextual information, while partial convolution is used to extract local features. This enables our model to capture both global and local features simultaneously, allowing it to detect subtle defects in the original images. As a result, our model outperforms the original Swin-Transformer in terms of both detection accuracy and speed.

**Figure 7:**
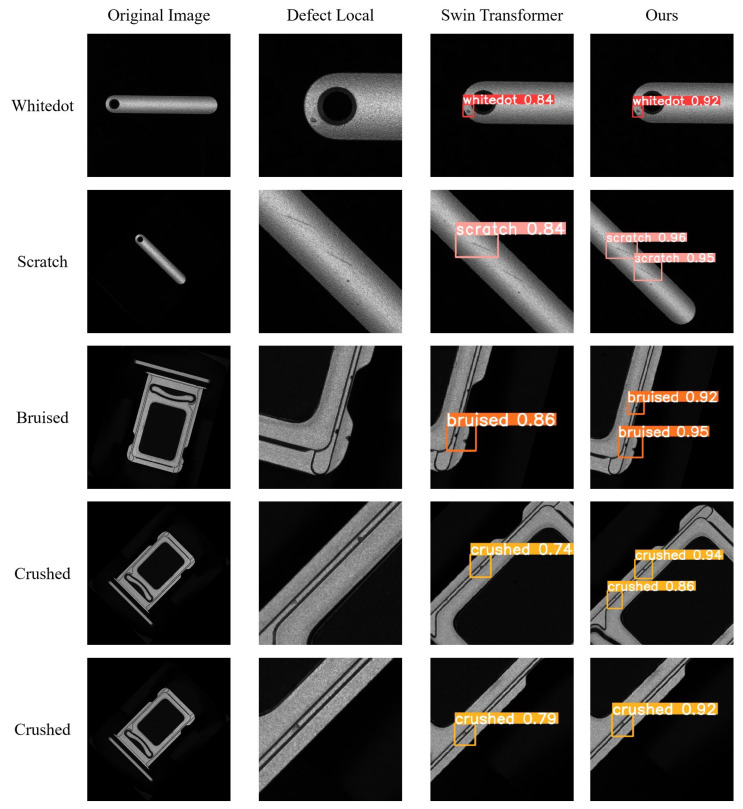
Comparison of detection results from different models on the SIM card slot dataset.

Figure 7 shows the detection effect of different models for different defects on the self-built SIM card slot defect dataset. As illustrated in Figure 7, the previous models have the problem of missing detection, while our improved model is able to detect defects more accurately at each location. Additionally, the confidence scores of our model are generally higher than the other models. This indicates that our model is more effective in capturing the textures and characteristics of defects in industrial products. In contrast, previous models with complex structures and feature extraction methods do not succeed in attaining competitive performance when it comes to detecting defects in industrial products.

#### 4.3.2. Experiments on the PKU-Market-PCB Dataset

To validate the effectiveness of our improvements and assess the generalization and robustness of our model on new data, we conduct similar experiments on the PKU-Market-PCB dataset. To ensure consistency, we use the same hyperparameters and data preprocessing methods. The results are presented in Table 2, where our model again achieved the best performance. Compared to the original Swin-Transformer, our model outperforms it by 1.7% in mAP and 51 FPS on this dataset. Also, the FLOPs of our model are decreased by 4.1 G.

**Figure 8:**
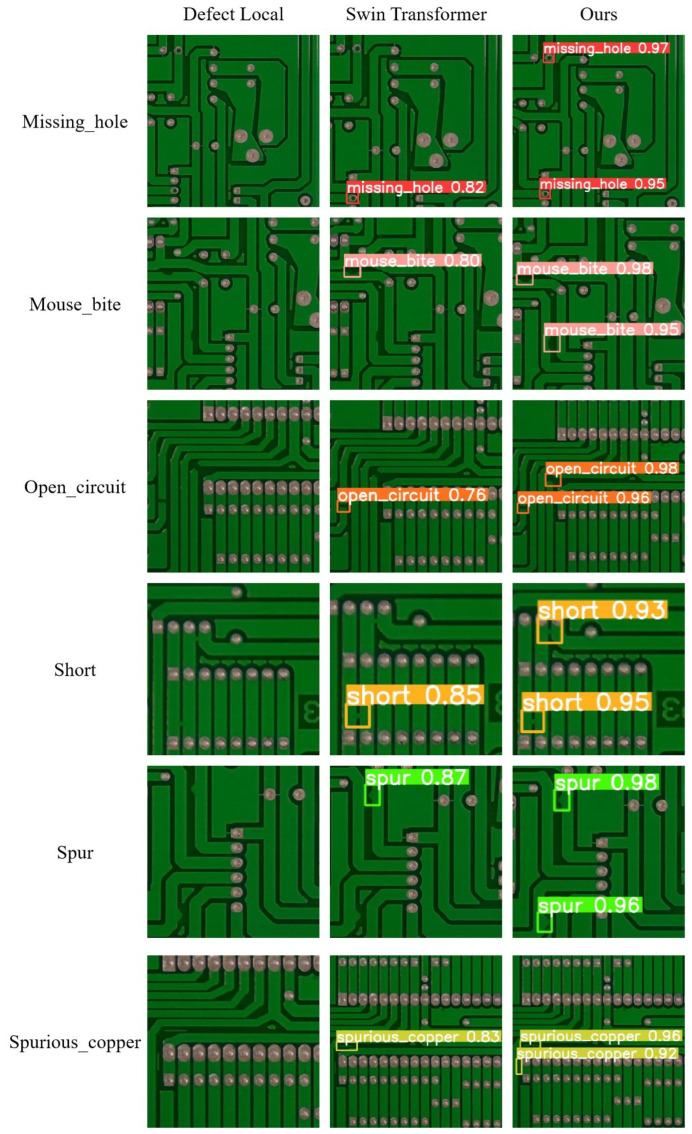
Comparison of the detection performance of various models on the PKU-Market-PCB dataset.

Figure 8 shows the detection results of different models for six types of defects on the PKU-Market-PCB dataset. Similar to the performance on the self-built SIM card slot defect dataset, the original Swin-Transformer and others models also suffers from missed detections. In contrast, the improved model successfully detects all defects with higher confidence.

### 4.4. Ablation Study

Ablation study is conducted on the two datasets to investigate the impact of pyramid pooling, linear attention, and partial convolution on the performance of the model. Table 3 and Table 4 present the effects of different modules on the model’s performance on these two datasets, respectively.

As shown in Table 3 and Table 4, both pyramid pooling and linear attention are effective in reducing the computational resources required for self-attention. However, this leads to a decrease in detection accuracy, particularly when both pyramid pooling and linear attention are used simultaneously, resulting in a significant decrease in performance. This is due to the characteristics of max pooling and linear attention. While max pooling emphasizes the most prominent features within a region, it inevitably discards some detailed information. It is difficult for linear attention to focus its distribution of attention as well as softmax attention, which weakens its local feature extraction ability, and therefore can lead to the missed detection of defects. By using partial convolution to aggregate neighboring feature tokens and fuse multi-scale feature information, the model captures global context without significantly increasing computational cost, while also enhancing local detail features. The experimental results on both datasets strongly validate the effectiveness of our improvements.

**Figure 9:**
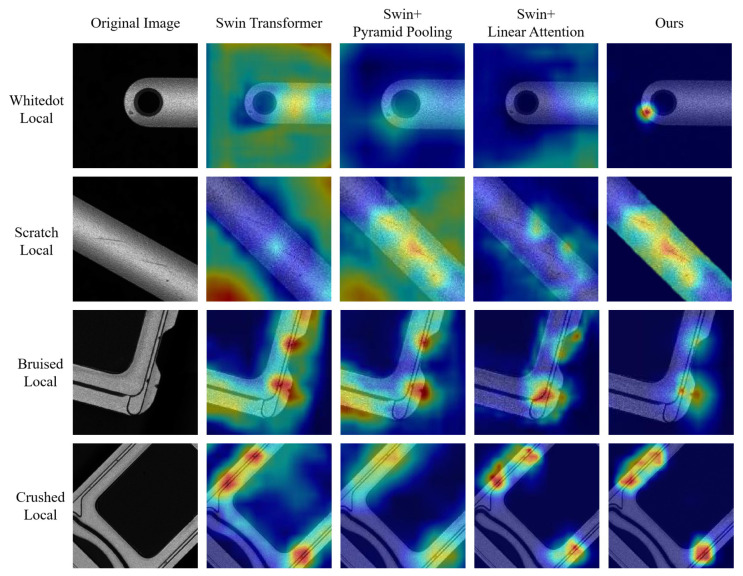
Comparison of heatmaps from different models on the SIM card slot dataset.

To further demonstrate the effectiveness of our algorithm, we present heatmaps for the original SIM card slot images, the Swin-Transformer model, the Swin-Transformer combined with pyramid pooling, the Swin-Transformer combined with linear attention, and our model, as shown in Figure 9. The columns in the figure correspond to the following: zoomed-in details of the original image, the heatmap of the Swin-Transformer, the heatmap of the Swin-Transformer with pyramid pooling, the heatmap of the Swin-Transformer with linear attention, and the heatmap of our model.

As shown in Figure 9, the Swin-Transformer model fails to focus on the defects in the image and instead places excessive attention on the background. After combining Swin-Transformer with pyramid pooling, this issue is partially improved, but the model still overemphasizes the background and neglects the defect locations. When combined with linear attention, the Swin-Transformer reduces its attention to the background, but it still does not correctly focus on the defect locations. In contrast, our model’s focus on the location of the defect basically coincides with the center of the defect, and basically focuses on the vicinity of the defect without deviation. This indicates that our improved model better captures the regions containing defects, demonstrating that our approach is both accurate and efficient.

**Figure 10:**
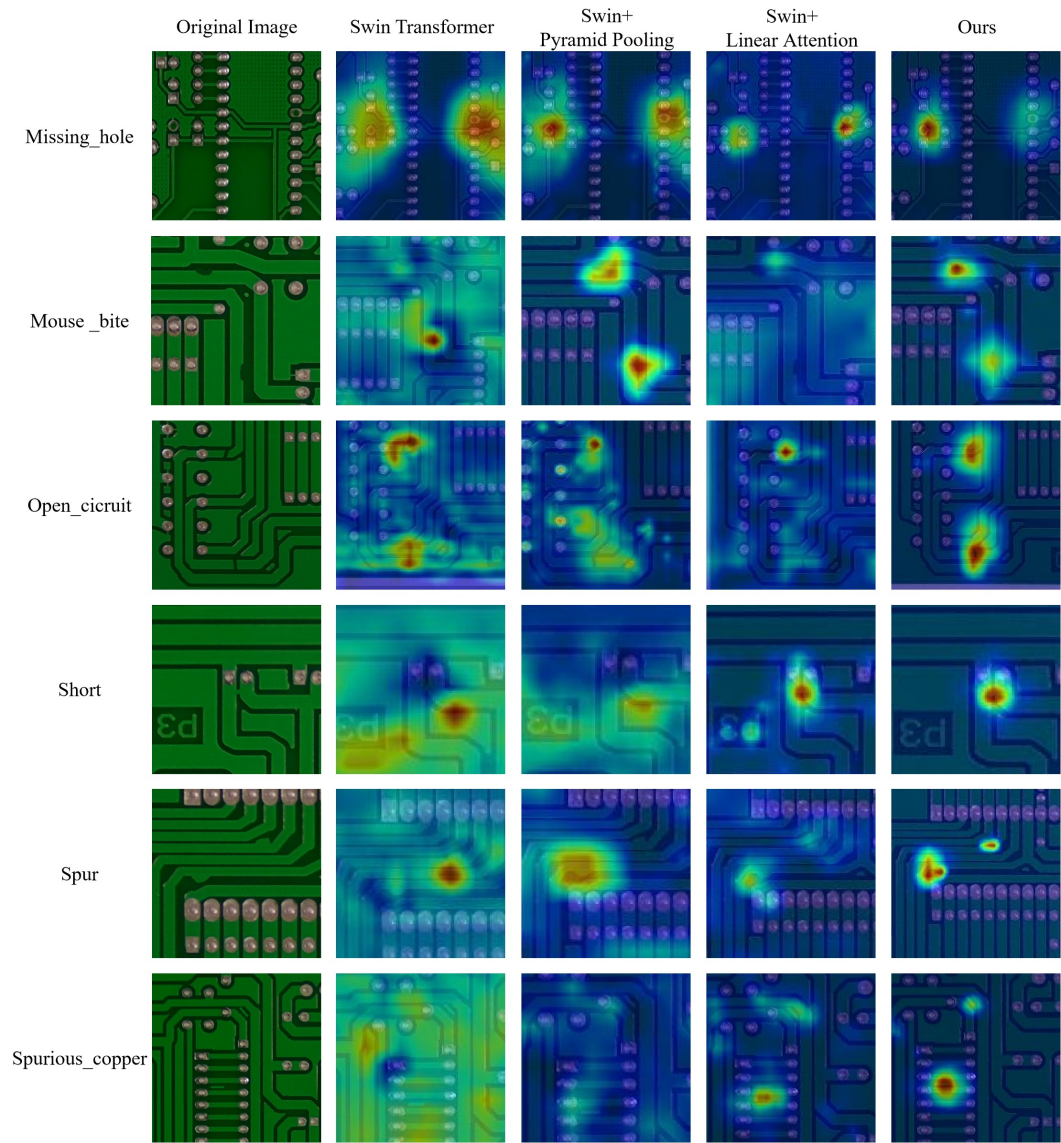
Comparison of heatmaps from different models on the PKU-Market-PCB dataset.

Figure 10 shows the heatmap results on the PKU-Market-PCB dataset. The original Swin-Transformer, Swin-Transformer with pyramid pooling, and Swin-Transformer with linear attention, individually applied, exhibit some redundancy or deviation in focusing on defect locations, along with unnecessary attention to the background. In contrast, our method precisely focuses on the defect area.

## 5. Conclusions

This paper addresses the issues of low detection accuracy and efficiency in existing algorithms for detecting subtle surface defects on industrial products. We improve the multi-head attention mechanism in the Swin-Transformer model by introducing a linear attention mechanism based on pyramid pooling. Pyramid pooling reduces the computational load of the multi-head attention mechanism. At the same time, softmax attention in the Swin-Transformer model is replaced with a simple linear attention mechanism, which further effectively reduces the demand for computing resources. In addition, partial convolution is introduced to make the model focus on subtle defect parts and improve the accuracy of defect detection. Experiments on self-built SIM card slot defect dataset and public PKU-Market-PCB dataset prove that the proposed algorithm can not only greatly improve the detection efficiency but also improve the detection accuracy. The proposed method effectively considers both detection efficiency and accuracy, thereby fulfilling the efficiency requirements for industrial defect detection. In the future, we aim to validate the algorithm’s feasibility using a broader range of datasets from real industrial products and enhance the model accordingly. However, the defect of this method is its limited effectiveness in object detection within natural scenes. Moving forward, we should focus on improving this model to enable it to adapt to a wider array of application scenarios.

## Figures and Tables

**Table 1:** The performance of our model and mainstream object detection models on the self-built SIM card slot defect dataset.

Model	FLOPs/G	mAP@IoU = 0.5/%	FPS
Faster R-CNN	28.7	88.1	11
YOLO-v8	27.6	91.2	29
P2T	26.8	89.5	35
EfficientViT	26.1	88.2	37
Swin Transformer	29.6	91.4	16
Ours	25.3	92.6	68

**Table 2:** Performance of our model and mainstream object detection models on the PKU-Market-PCB dataset.

Model	FLOPs/G	mAP@IoU = 0.5/%	FPS
Faster R-CNN	24.0	92.3	12
YOLO-v8	22.7	93.9	27
P2T	22.4	93.5	36
EfficientViT	22.1	92.3	35
Swin Transformer	24.9	95.4	18
Ours	20.8	97.1	69

**Table 3:** The impact of different modules on the model’s performance of the self-built SIM card slot defect dataset.

Pyramid Pooling	Linear Attention	Partial Convolution	FLOPs/G	mAP@IoU = 0.5/%	FPS
			29.6	91.4	16
✓			27.2	90.9	36
	✓		27.0	90.3	39
✓	✓		24.6	89.8	71
✓	✓	✓	25.3	92.6	68

**Table 4:** The impact of different modules on the model’s performance of the PKU-Market-PCB dataset.

Pyramid Pooling	Linear Attention	Partial Convolution	FLOPs/G	mAP@IoU = 0.5/%	FPS
			24.9	95.4	18
✓			22.6	94.6	36
	✓		22.4	94.1	38
✓	✓		20.1	93.5	70
✓	✓	✓	20.8	97.1	69

## Data Availability

Data are contained within the article.
